# Obesity paradox for critically ill patients may be modified by age: a retrospective observational study from one large database

**DOI:** 10.1186/s13054-020-03157-1

**Published:** 2020-07-13

**Authors:** Dawei Zhou, Zhimin Li, Guangzhi Shi, Jianxin Zhou

**Affiliations:** Department of Critical Care Medicine, Beijing Tiantan Hospital, Capital Medical University, Beijing, China

To the Editor:

“Obesity paradox,” the phenomenon that obesity increases the risk of obesity-related diseases but paradoxically is associated with a survival benefit, has been observed in some researches related to critical illnesses [1, 2]. However, the pathophysiologic mechanisms of the obesity survival paradox are currently conjecture. An observational study with a 12-year follow-up in the general population reported that the relative risk for mortality associated with an increased body mass index (BMI) declined with age [3]. However, the short-term effect of age on the association between BMI and in-hospital mortality for critically ill patients is unknown. We therefore aimed to evaluate the effect of age on the “obesity paradox” in an ICU population.

We included all adult patients from the eICU Collaborative Research Database (eicu-crd.mit.edu) that had a first admission to the ICU and whose BMI was > 10 and < 70 kg/m^2^ [4]. Obesity was assessed as a six-category variable according to BMI [5]: underweight, BMI < 18.5 kg/m^2^; normal weight, 18.5 ≤ BMI < 25 kg/m^2^; overweight, 25 ≤ BMI < 30 kg/m^2^; obesity grade 1, 30 ≤ BMI < 35 kg/m^2^; obesity grade 2, 35 ≤ BMI < 40 kg/m^2^; and obesity grade 3, BMI ≥ 40 kg/m^2^. The primary endpoint was in-hospital mortality. Hospital mortality was considered as a time-to-event variable. Patients were censored when discharged alive. We used Cox regression analysis to produce adjusted hazard ratios (HRs) for the association between BMI and hospital mortality. The confounders included gender, ethnicity, comorbidities, ICU types, and acute physiology score. BMI was examined as both a categorical and continuous variable. The linear trend for hazard ratio with an increase of 1.0 in BMI across age groups was tested by Cox regression analysis with inverse-variance weighted average. Multiple imputation was used to deal with the missing data.

The final cohort included 148,783 ICU patients, with in-hospital mortality of 9% (Table 1). 54,572 (36.7%) patients were classified as obese (BMI ≥ 30 kg/m^2^). As expected, higher BMI categories had a higher percentage of comorbidities of hypertension, diabetes mellitus, chronic heart failure, and chronic kidney disease. Higher BMI was associated with lower ICU and in-hospital mortality.

**Table 1:** Baseline and clinical characteristics of study patients by BMI categories

Variables	Total	Underweight (BMI < 18.5 kg/m^2^)	Normal weight (18.5 kg/m^2^ ≤ BMI < 25 kg/m^2^)	Overweight (25 kg/m^2^ ≤ BMI < 30 kg/m^2^)	Obesity grade 1 (30 kg/m^2^ ≤ BMI < 35 kg/m^2^)	Obesity grade 2 (35 kg/m^2^ ≤ BMI < 40 kg/m^2^)	Obesity grade 3 (BMI ≥ 40 kg/m^2^)
Number of patients	148,783 (100%)	6328 (4.3%)	43,960 (29.5%)	43,923 (29.5%)	27,584 (18.5%)	13,881 (9.3%)	13,107 (8.8%)
Age							
Median, years	65 (53,76)	67 (54,80)	67 (53,80)	66 (54,77)	64 (53,74)	62 (52,72)	60 (49,68)
≤ 30	8348 (6)	506 (8)	3530 (8)	2107 (5)	1082 (4)	544 (4)	579 (4)
31–40	8683 (6)	314 (5)	2503 (6)	2399 (5)	1547 (6)	891 (6)	1029 (8)
41–50	14,947 (10)	467 (7)	3801 (9)	4056 (9)	2963 (11)	1712 (12)	1948 (15)
51–60	27,739 (19)	1086 (17)	6836 (16)	7847 (18)	5519 (20)	3119 (22)	3332 (25)
61–70	33,442 (22)	1193 (19)	8304 (19)	9728 (22)	6933 (25)	3698 (27)	3586 (27)
71–80	30,823 (21)	1277 (20)	8604 (20)	9926 (23)	6179 (22)	2790 (20)	2047 (16)
> 80	24,801 (17)	1485 (23)	10,382 (24)	7860 (18)	3361 (12)	1127 (8)	586 (4)
Gender: male	80,294 (54)	2669 (42)	23,526 (54)	26,247 (60)	15,617 (57)	6973 (50)	5262 (40)
BMI, kg/m^2^	27.5 (23.5, 32.7)	17.1 (16, 17.9)	22.4 (20.9, 23.7)	27.3 (26.1, 28.5)	32.1 (30.9, 33.4)	37 (35.9, 38.3)	44.7 (41.9, 49.6)
Ethnicity							
Caucasian	114,199 (77)	4766 (75)	33,565 (76)	33,786 (77)	21,462 (78)	10,696 (77)	9924 (76)
African American	16,537 (11)	837 (13)	4597 (10)	4364 (10)	2975 (11)	1753 (13)	2011 (15)
Hispanic	5763 (4)	197 (3)	1754 (4)	1909 (4)	1034 (4)	467 (3)	402 (3)
Asian	2494 (2)	191 (3)	1146 (3)	745 (2)	277 (1)	85 (1)	50 (0)
Native American	1090 (1)	33 (1)	290 (1)	303 (1)	198 (1)	128 (1)	138 (1)
Others/unknown	8700 (6)	304 (5)	2608 (6)	2816 (6)	1638 (6)	752 (5)	582 (4)
Comorbidities							
Hypertension	74,964 (50)	2536 (40)	19,663 (45)	22,171 (50)	15,088 (55)	7887 (57)	7619 (58)
Diabetes mellitus	43,420 (29)	1058 (17)	9333 (21)	11,840 (27)	9432 (34)	5620 (40)	6137 (47)
Stroke	12,315 (8)	590 (9)	3920 (9)	3716 (8)	2195 (8)	1015 (7)	879 (7)
Tumor	22,356 (15)	1295 (20)	7465 (17)	6674 (15)	3736 (14)	1772 (13)	1414 (11)
Respiratory disease	22,912 (15)	1565 (25)	6733 (15)	5736 (13)	3838 (14)	2252 (16)	2788 (21)
CHF	21,868 (15)	734 (12)	5683 (13)	5904 (13)	4164 (15)	2463 (18)	2920 (22)
Cirrhosis	4144 (3)	175 (3)	1308 (3)	1224 (3)	755 (3)	362 (3)	320 (2)
CKD	18,134 (12)	663 (10)	5131 (12)	5186 (12)	3441 (12)	1813 (13)	1900 (14)
ICU types							
Med-SurgICU	82,057 (55)	3724 (59)	24,922 (57)	23,652 (54)	14,738 (53)	7519 (54)	7502 (57)
Cardiac ICU	10,711 (7)	511 (8)	3047 (7)	3148 (7)	2028 (7)	1000 (7)	977 (7)
CCU-CTICU	12,555 (8)	354 (6)	3186 (7)	3894 (9)	2655 (10)	1371 (10)	1095 (8)
CSICU	5528 (4)	183 (3)	1492 (3)	1802 (4)	1114 (4)	531 (4)	406 (3)
CTICU	4685 (3)	138 (2)	1246 (3)	1533 (3)	1032 (4)	457 (3)	279 (2)
MICU	12,758 (9)	677 (11)	3861 (9)	3566 (8)	2163 (8)	1190 (9)	1301 (10)
Neuro ICU	11,245 (8)	357 (6)	3445 (8)	3457 (8)	2182 (8)	1003 (7)	801 (6)
SICU	9244 (6)	384 (6)	2761 (6)	2871 (7)	1672 (6)	810 (6)	746 (6)
ICU scoring systems							
SOFA score	4 (2, 7)	5 (2, 7)	4 (2, 7)	4 (2, 6)	4 (2, 6)	4 (2, 7)	4 (2, 7)
APS	38 (27, 53)	42 (30, 59)	38 (28, 54)	37 (26, 52)	36 (26, 52)	37 (26, 53)	39 (27, 55)
APACHE IV score	50 (37, 68)	56 (42, 74)	52 (38, 69)	50 (37, 67)	49 (36, 66)	49 (35, 66)	49 (36, 67)
ICU LOS, days	2 (1, 4)	2 (1, 4)	2 (1, 4)	2 (1, 4)	2 (1, 4)	2 (1, 4)	2 (1, 4)
Hospital LOS, days	5 (3, 8)	5 (3, 9)	5 (3, 8)	5 (3, 8)	5 (3, 8)	5 (3, 9)	5 (3, 9)
ICU mortality	7938 (5)	506 (8)	2592 (6)	2182 (5)	1269 (5)	705 (5)	684 (5)
Hospital mortality	12,867 (9)	897 (14)	4289 (10)	3536 (8)	2032 (7)	1064 (8)	1049 (8)

Data are median (interquartile range) or no./total (%)

*BMI* body mass index, *CHF* chronic heart failure, *CKD* chronic kidney disease, *ICU* intensive care unit, *CCU* coronary care unit, *CTICU* cardiothoracic ICU, *CSICU* cardiac surgery ICU, *MICU* medical ICU, *SICU* surgical ICU, *SOFA* Sequential Organ Failure Assessment, *APS* Acute Physiology Score, *APACHE* Acute Physiology and Chronic Health Evaluation, *LOS* length of stay

When considering normal weight patients as the reference, underweight patients had higher HR for in-hospital mortality, while overweight and obesity patients had lower HRs (Fig. 1A). After adjusting for confounders, the association of BMI with mortality was not significant for age ≤ 30, 31–40, and 41–50 years groups, while mortality was significantly lower with higher BMI for age 51–60, 61–70, 71–80, and ≥ 80 years groups (Fig. 1B, *P* for trend < 0.001).

**Fig. 1:**
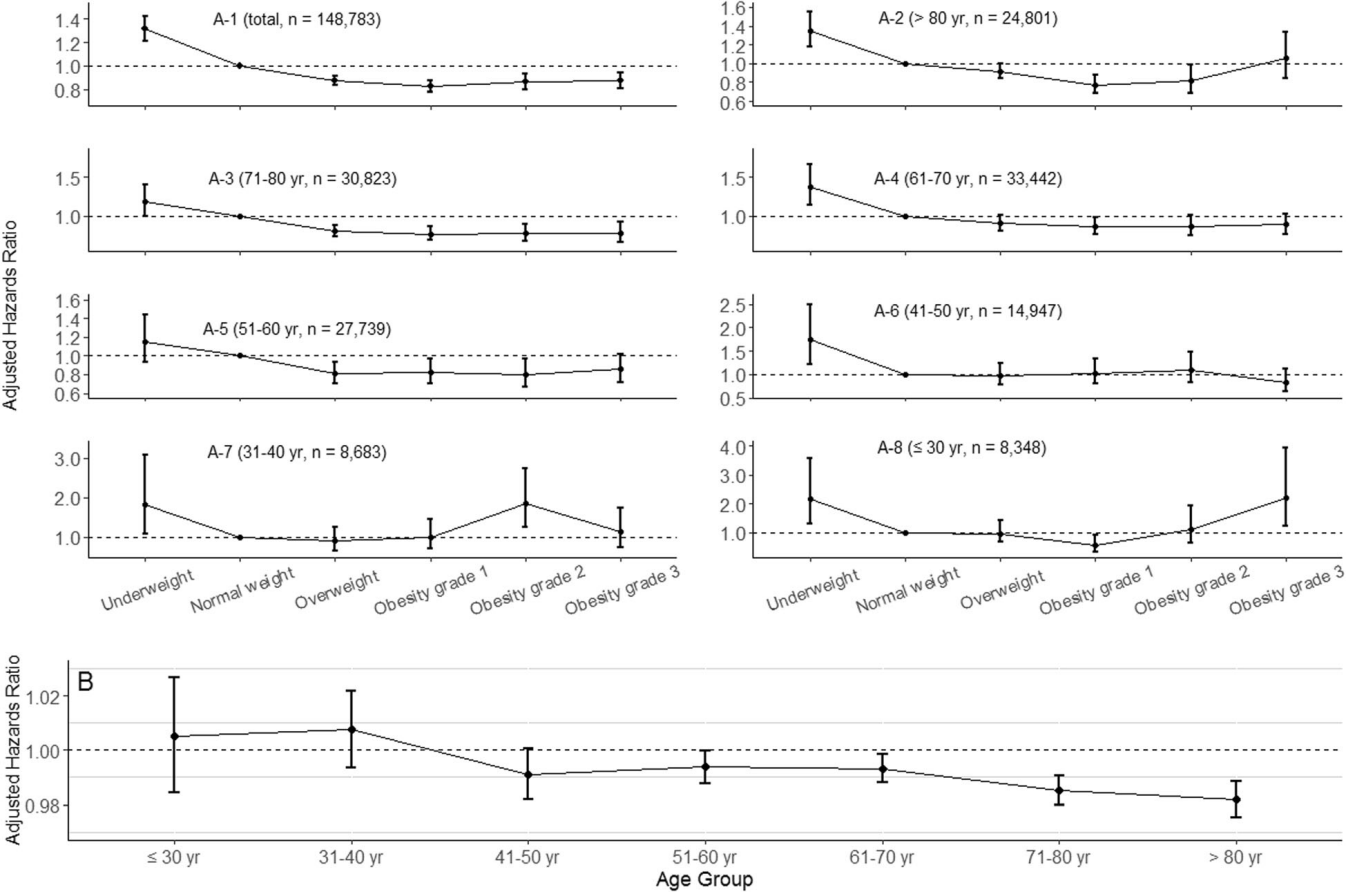
**A1**–**8** Adjusted hazard ratio for in-hospital mortality according to age group and obesity categories. All hazard ratios were adjusted for gender, ethnicity, comorbidities, ICU types, and acute physiology score with Cox proportional hazard models. Normal weight was considered as the reference category. The bars represented 95% confidence interval. **B** Adjusted hazard ratio for in-hospital mortality associated with an increase of 1.0 kg/m^2^ body mass index. Hazard ratios were from separate Cox proportional hazard models tested within the different age groups, with body mass index entered as a continuous variable. All hazard ratios were adjusted for gender, ethnicity, comorbidities, ICU types, and acute physiology score. The test for trend was significant for in-hospital mortality (*P* < 0.001)

The obesity paradox has been hypothesized to result from the extra fat tissue functioning as a fuel source or from the immunomodulatory effects of substances secreted by fat cells [2, 6]. The age effects could be attributed to older patients are more at risk of malnutrition. Whether the endocrine role of fat in critical illness differs with age has not been investigated. Overall, the potential mechanisms are unclear, which needs further research. The results should be interpreted with caution because of the limitations, such as the retrospective nature, the risk of residual confounding, and the missing data of BMI. The prevalence of obesity is increasing, also in the ICU population [2], and acknowledging the differential impact of obesity on mortality according to age class may help to improve outcome prediction.

In conclusion, our data are in concert with the obesity paradox and suggest that age has a modifying effect on the association between BMI and in-hospital mortality. Overweight or obesity may be more beneficial for older adult critically ill patients. Further study is needed to investigate the potential mechanisms.

## Data Availability

Data analyzed during the present study are currently stored in the eICU database (eicu-crd.mit.edu). After completing the required training course (the Collaborative Institutional Training Initiative) and requesting access to the eICU Collaborative Research Database, researchers can seek to use the database.

## Abbreviations

BMI: Body mass index
HR: Hazard ratio
ICU: Intensive care unit
